# Prognostic Value of Coronary Artery Calcification Identified by the Semi-quantitative Weston Method in the Emergency Room or Other Hospitalized Patients

**DOI:** 10.3389/fcvm.2021.684292

**Published:** 2021-06-16

**Authors:** Lu Chen, Andrey Vavrenyuk, Jun Hong Ren, Pankil Desai, Joseph Bahgat, Michael A. Bernstein, Michael I. Ebright, Mamatha Gowda, Suzanne Rose, Arzhang Fallahi, Brian Stainken, David H. Hsi

**Affiliations:** ^1^Department of Ultrasound, Shanghai Chest Hospital, Shanghai Jiao Tong University, Shanghai, China; ^2^Heart and Vascular Institute, Stamford Hospital, Stamford, CT, United States; ^3^Department of Medicine, Stamford Hospital, Stamford, CT, United States; ^4^Division of Pulmonary Medicine and Critical Care, Stamford Hospital, Stamford, CT, United States; ^5^Division of Thoracic Surgery, Stamford Hospital, Stamford, CT, United States; ^6^Department of Radiology, Stamford Hospital, Stamford, CT, United States; ^7^Office of Research, Stamford Hospital, Stamford, CT, United States

**Keywords:** coronary artery calcification, non-gated chest CT, Weston method for CAC, adverse cardiac events, all-cause mortality

## Abstract

**Background:** Coronary artery calcification (CAC) may provide insight to the patients' coronary artery disease (CAD) risks and influence early intervention. With increasing use of non-gated CT scans in clinical practice, the visual coronary artery scoring system (Weston Method) could quickly provide clinicians with important information of CAC for patient triage and management.

**Methods:** We retrospectively studied the available CT imaging data and estimated CAC burden using the Weston method in 493 emergency room or other hospitalized patients. The Weston scores were calculated by the sum of the score for each vessel including the left main, left anterior descending, left circumflex artery and right coronary artery (range 0–12). The primary endpoint was a composite of the major adverse cardiac events (MACEs), including cardiac death, myocardial infarction, stroke, and coronary revascularization.

**Results:** During a median follow-up of 85 months, a total of 25 (5.1%) MACE were recorded and 57 (11.2%) patients died from any causes. Detectable CAC was most common (96%) in the left anterior descending coronary arteries. Multivariable analysis showed that CAC total scores were independent predictors for MACE and all-cause mortality. Receiver operating characteristic analysis showed that CAC total score ≥5 was the optimal cutoff value for predicting MACEs.

**Conclusions:** In the emergency room and hospitalized patients, the semi-quantitation of CAC burden using the Weston score system was related to the long-term cardiovascular outcomes including mortality. Clinicians and radiologists should maximize the value of non-contrast chest CT images by reporting CAC details.

## Introduction

Coronary artery calcium (CAC) is a highly specific marker for overall plaque burden of coronary atherosclerosis and correlates well with an integration of all the risk factors over the lifetime of an individual, which plays important roles for primary prevention of cardiac events (1). CAC score on multi-detector computed tomography (CT), an imaging technique used to non-invasively quantify coronary calcium, has become a robust method in predicting cardiovascular disease (CAD) risk and serious cardiac events leading to mortality (2). It has been found that CAC score performs better than other risk assessment tools to identify those asymptomatic patients that would benefit from medical therapies (2, 3).

As the traditional CAC scoring method, the Agatston method has been widely used in clinical practice (4, 5). But due to very limited insurance coverage for CAC scanning, some patients at risk for CAD are not able to benefit from dedicated ECG-gated CAC evaluation. Therefore, clinicians have been focusing on finding an alternative CAC scoring method to bring some cost efficiency to patients. With the widespread application of standard non-contrast chest CT for emergency room and admitted patients, a pioneering study from the Cleveland Clinic reported that visual coronary artery scoring system (Weston Method) on standard non-contrast chest CT correlated well with the Agatston Method (6). Radiologists or cardiologists could easily visualize coronary artery calcium on non-contrast chest CT providing insight to the patients' coronary status and influencing the early decision-making process. However, the evidence on the relationship between the Weston score and long-term prognosis is scarce, and the reports of non-contrast chest CT lacks a description of CAC with location, extent, or severity.

This study attempted to evaluate the value of the Weston score in predicting adverse outcome in those presenting to the emergency room or other hospitalized patients undergoing standard non-contrast chest CT for any indication, thereby encouraging radiologists to describe coronary calcification in detail.

## Methods

### Study Design and Patient Population

We performed a retrospective review of 493 patients consecutively referred for standard non-contrast chest CT examinations for any reason from January 2012 to November 2014. This investigation was approved by the institutional review board of Stamford Hospital (Quorum Review Institutional Review Board QR# 32130).

We included patients older than 18 years and younger than 80 years of age. Exclusion criteria were (i) patients with a known history of prior coronary revascularization, acute coronary syndrome, heart failure and valve replacement, (ii) patients with inadequate image quality due to significant respiratory motion artifacts, (iii) patients with missing follow-up data. Information on patient demographics and clinical conditions were collected and analyzed.

### CT Examinations and Reading of the Images

All studies were conducted on a 64-slice MDCT system (Canon Aquilion 64, Japan). The technical parameters of the acquisition were as follows: 120 kVp; 40–80 mAs (depending on weight); detector collimation, 1.25–2.5 mm; slice thickness 3 mm; reconstruction interval 3 mm; algorithm: body FC17, lung FC 56. The machine picked a variable mA along the patients' z-axis based off the scout. The scans were initially reviewed by experienced radiologists, with the final interpretation performed by two cardiologists. The readers viewed the images on a high-spatial resolution monitor at its typical window and level settings. The standard CT images were analyzed visually using mediastinum soft tissue window settings (window width 400, window length 40).

**Figure 1:**
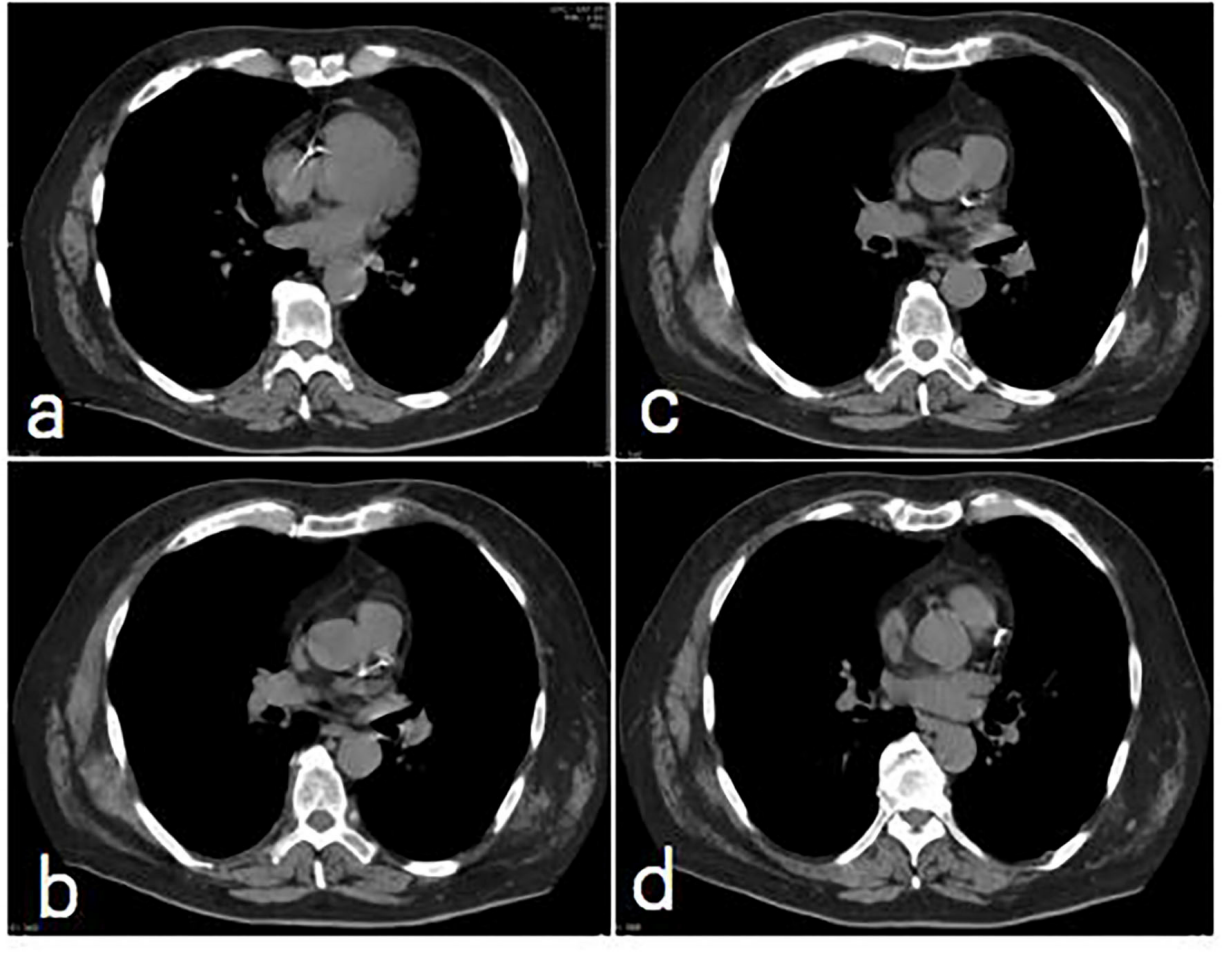
A non contrast, non-gated Chest CT image series for CAC **(a)** RCA calcification score = 3; **(b)** LM calcification score = 3; **(c)** mid-LAD score = 3; **(d)** proximal LAD calcification score = 3. The total CAC score = 9.

The Weston score assigns values based on visual estimates for the presence and degree of calcification in each major coronary vessel (the left main trunk, left anterior descending artery, left circumflex artery, and right coronary artery), as follows: (0): no visually detected calcium; (1): a single high-density pixel; (3): the calcium was dense enough to create blooming artifact; and (2): for calcium between 1 and 3 (6). The CAC score was calculated by the sum of the score for each vessel (range 0–12). All readers were blinded to the results of the participants' demographic and clinical data. Figure 1 showed illustrative CAC images in one patient who had subsequent cardiac death.

### Evaluation of Adverse Outcomes

The primary endpoint was major adverse cardiac events (MACEs) including cardiac death, non-fatal myocardial infarction (MI), non-fatal stroke, and coronary revascularization. Non-fatal MI was defined as an elevated high sensitivity troponin I with ischemic symptoms or electrocardiographic findings suggestive of ischemia. Stroke was defined as any focal or neurological deficit of abrupt onset lasting more than 24 h. Coronary revascularization was defined by percutaneous coronary intervention or coronary artery bypass surgery as documented in the electronic medical record.

The secondary endpoint was all-cause mortality. All in-house or subsequent deaths were directly confirmed in our patient data base. The cause of death was coded according to the International Classification of Diseases, 10th revision. Follow-up time was calculated from the time of chest CT to the time of death.

### Statistical Analysis

Continuous data were presented as the mean ± standard deviations, whereas categorical data are presented as percentages. Means were compared using the repeated measurement analysis of variance. Intra-observer and inter-observer agreements were calculated using the coefficient of variation (i.e., the percentage absolute difference between the measurements divided by their mean value) and the intra-class correlation coefficient. A Cox proportional hazards model was used in the univariable and multivariable analyses to investigate the association between adverse outcomes and clinical factors. A stepwise variable selection was performed in the multivariable analysis retaining all predicators with *P* < 0.05 in the final model. The hazard ratio (HR) and its associated 95% confidence interval (CI) were reported. Receiver operating characteristic (ROC) curve analysis was used to test the value of significant predictors of adverse outcomes. The cut-off value was selected as the value corresponding to the highest average of sensitivity and specificity, and then were analyzed using the Kaplan-Meier method. To estimate the significance of the Kaplan-Meier curves, the log-rank test was used. *P* < 0.05 were considered statistically significant. All the statistical analyses were performed using MedCalc for Windows, version 19.3 (MedCalc Software, Ostend, Belgium).

## Results

### Clinical Characteristics of Study Patients

The baseline characteristics of 493 subjects are shown in Table 1. The median age was 60 years (range, 35–79 years), 245 (49.7%) were female, 328 (66.5%) were white, 97 (19.7%) were black, 51 (10.3%) were Hispanic, and 17 (3.4%) were Chinese.

**Table 1:** Baseline characteristics for all patients with Events and no events.

	**Overall**	**Events**	**No events**	***P*-value**
	***n* = 493**	***n* = 67**	***n* = 426**	
Age	60.21 ± 12.41	66.65 ± 6.19	58.57 ± 12.65	0.003
Female, %	245 (49.7%)	25 (37.3%)	220 (51.6%)	0.029
White, %	324 (65.7%)	40 (59.7%)	284 (66.6%)	0.574
SBP, mmHg	131.93 ± 18.39	132.27 ± 19.19	131.88 ± 18.29	0.875
DBP, mmHg	72.72 ± 11.56	71.68 ± 13.84	73.18 ± 11.12	0.053
Troponin I >0.3 ug/L	37 (7.5%)	17 (25.4%)	20 (4.7%)	0.000
Diabetes, %	126 (25.6%)	27 (40.3%)	99 (23.2%)	0.002
Hyperlipidemia, %	84 (17.0%)	22 (32.8%)	172 (40.4%)	0.330
**Smoking status, %**				
Former	89 (18.0%)	19 (28.4%)	70 (16.4%)	0.061
Current	99 (20.1%)	37 (55.2%)	62 (14.6%)	0.001
Alcohol history, %	65 (13.2%)	13 (19.4%)	52 (12.2%)	0.083
Aspirin use, %	116 (23.5%)	22 (32.8%)	94 (22.1%)	0.053
Statin use, %	160 (32.5%)	22 (32.8%)	139 (32.6%)	0.943
CAC score	2.53 ± 3.48	5.58 ± 4.47	2.04 ± 3.03	0.000

*Data are presented as mean ± SD or No. (%). SBP, Systolic blood pressure; DBP, Diastole blood pressure; CAC, Coronary artery calcification. “Event” group included all death and major adverse cardiovascular events. The “Events” group had total of 67 patients: all-cause death (including 15 cardiac deaths) in 57 patients and 25 major adverse cardiovascular events [57 + (25–15)] = 67 total*.

CT coronary calcium analysis was analyzed in all 493 patients. However, among the original 550 participants screened, we had to exclude 57 subjects who had poor image quality such as motion artifact. Thus, the true success rate of Weston method of CAC scoring was actually 89.6%. Main indications for chest CT included: shortness of breath (252, 51.1%); lung mass evaluation (115, 23.3%); chest pain (60, 12.2%); pulmonary infiltrates (18, 3.7%); cough (29, 5.9%), pneumonia (1); hemoptysis (2); status post cardiac arrest (3); fall/trauma (4); unclear indications (7).

A total of 262 patients (53.1%) had visible CAC. Among them, 96 (19.5%) had one-vessel CAC; 114 (23.1%) had 3-vessel CAC, 89 (18.1%) and 173 (35.1%) affected the left main (LM) and left anterior descending (LAD) coronary arteries, respectively. Traditional risk factors were equally distributed in the two categories of having adverse outcomes (events) and no events. Significant differences were found between the two groups, with older age, male, diabetes, current smoking status and higher mean CAC scores in the events group (Table 1). The chest CTs were obtained in the emergency room (250, 50.7%), in the hospital (73, 14.8%), and at the outpatient location with subsequent hospitalizations (161, 32.7%). The CAC positive rate for the emergency room patients was 63.6%; inpatients 49.3%; and outpatients with subsequent hospitalization 54.0%.

### Follow Up

The median follow-up period was 85 months (interquartile range, 13 months). During the follow-up, 25 (5.1%) MACEs were recorded including 6 non-fatal MI, 15 cardiac death, 2 non-fatal stroke, and 2 myocardial revascularization procedures.

A total of 57 (11.6%) deaths for any reason occurred. The average age of death was 63 years, 61.5% were male; 36 subjects (55.4%) were not on statin drugs or aspirin. Among 231 patients with a CAC score of zero, 11 patients died of non-cardiac reason and one died of cardiac event due to non-ischemic heart failure.

The CAC findings in 25 patients with MACE showed total CAC score 8.72 ± 3.66, LAD score 2.8 ± 0.71, LM score 1.72 ± 1.34, LCX score 1.96 ± 1.34, and RCA score 2.24 ± 1.13. The detailed CAC findings in patients with MACE was listed in Table A1.

### Association Between CAC Score and Adverse Outcomes

Among the patients with MACEs, 23/25 (92%) had visible CAC and 16/25 (64%) had ≥3-vessel CAC, and detectable CAC was most common (96%) in the left anterior descending coronary arteries. By comparison, 60, 80, and 84% of patients had detectable CAC in the left main, left circumflex and right coronary arteries in the MACEs group, respectively. For the patients with all-cause death, 46/57 (81.5%) had visible CAC and 28/57(49.1%) had ≥3-vessel CAC.

Multivariate models demonstrated that CAC total score was a significant predictor for MACEs (HR 1.30, 95% CI 1.16–1.45, *P* < 0.0001) and all-cause mortality (HR 1.11, 95% CI 1.14–1.69, *P* < 0.0001) after adjusting for age, gender, ethnicity, hypertension, hyperlipidemia, diabetes, smoking, and statin use (Table 2).

**Table 2:** Multivariable Cox regression analysis for adverse outcomes according to CAC total score.

**Mode**	**HR (95% CI)**	***P*-value**
**MACE**		
Model 1 (unadjusted)	1.44 (1.30, 1.59)	<0.0001
Model 2 (age, sex, race)	1.41 (1.27, 1.57)	<0.0001
Model 3 (age, sex, race, HTN, HPL, DM)	1.39 (1.25, 1.56)	<0.0001
Model 4 (age, sex, race, HTN, HPL, DM, TnI)	1.32 (1.18, 1.49)	<0.0001
Model 5 (age, sex, race, HTN, HPL, DM, TnI, smoking)	1.32 (1.17, 1.48)	<0.0001
Model 6 (age, sex, race, HTN, HPL, DM, TnI, smoking, statin use)	1.31 (1.17, 1.48)	<0.0001
**All-cause mortality**		
Model 1 (unadjusted)	1.21 (1.15, 1.28)	<0.0001
Model 2 (age, sex, race)	1.19 (1.12, 1.27)	<0.0001
Model 3 (age, sex, race, HTN, HPL, DM)	1.19 (1.12, 1.27)	<0.0001
Model 4 (age, sex, race, HTN, HPL, DM, TnI)	1.16 (1.08, 1.24)	<0.0001
Model 5 (age, sex, race, HTN, HPL, DM, TnI, smoking)	1.12 (1.05, 1.20)	0.001
Model 6 (age, sex, race, HTN, HPL, DM, TnI, smoking, statin use)	1.12 (1.05, 1.20)	0.001

*HR, Hazard ratio; CI, Confidence interval; MACE, Major adverse cardiovascular events; HTN, Hypertension; HPL, Hyperlipidemia; DM, Diabetes Mellitus; TnI, Troponin I*.

Our data showed positive CAC in about 50% of patients who had chest CTs for any reason, but much higher percentage in patients with chest pain (61.7%) and in the emergency room 63.6%. These patients may gain more benefit from the detailed analysis for coronary artery calcification form the chest CT data.

**Figure 2:**
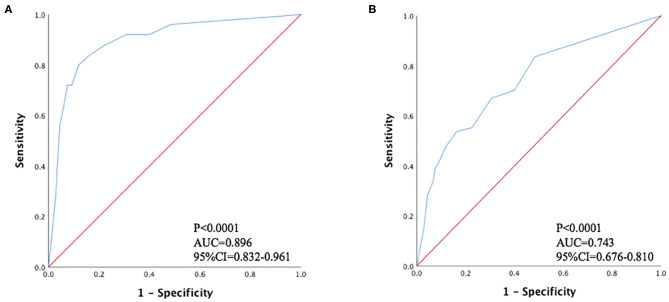
Receiver operating characteristic curves for predicting **(A)** major adverse cardiovascular events; **(B)** all-cause mortality.

**Figure 3:**
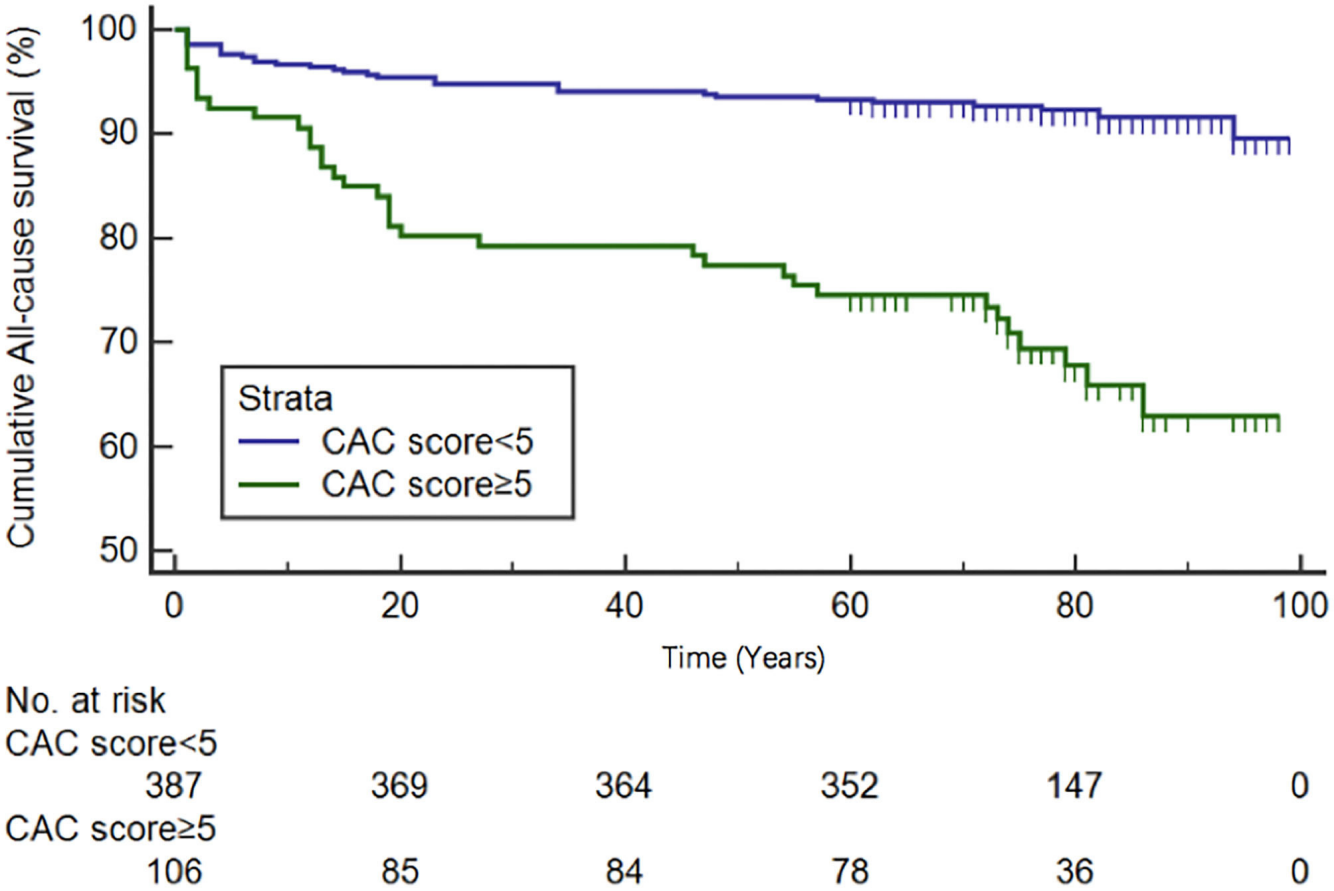
Kaplan-Meier overall survival curves for patients dichotomized by CAC total score ≥ or <5.

Based on ROC analysis, CAC total score ≥5 was the optimal cutoff value for predicting MACEs (AUC 0.896, sensitivity 84.0%, specificity 83.6%, 95% CI 0.830–0.963, *P* < 0.0001) and all-cause mortality (AUC 0.743, sensitivity 53.7%, specificity 83.6%, 95% CI 0.676–0.810, *P* < 0.0001) (Figure 2). The CAC total score data ranked according to the ROC analysis was used for overall survival (OS) estimation via the Kaplan-Meier method. Figure 3 show the OS estimates for patients stratified by CAC total score cut-off values. The differences in OS were statistically significant (log-rank test, *P* < 0.0001).

### Intra-observer and Inter-observer Variability

The intra-observer and inter-observer variabilities for CAC scores were 4.2 ± 1.7% and 5.5 ± 1.6%, respectively. The interclass correlation coefficients were 0.94 and 0.92 for intra-observer and inter-observer CAC score assessment, respectively.

## Discussion

In this retrospective single center study, we found that Weston score assessment of the CAC on non-gated chest CT provided independent prognostic value beyond traditional cardiovascular risk factors for the patients presenting to the emergency room as well as hospitalized patients. The Weston score could be potentially performed during all routine chest CT reporting, given that it may improve risk stratification and early intervention. Our results provided evidence to support the expanded reports of routine chest CT scan in emergency room and inpatients to routinely include CAC assessment. Due to the simplicity of this method, it can be easily adopted by radiologists and non-imaging clinical team members.

Many studies have confirmed that the Agatston scoring system, as the most commonly used method for detecting coronary calcification, can predict long-term adverse outcomes (8, 9). However, due to economic reasons, not all patients are eligible for this test. Clinicians have been looking for an affordable and convenient alternative scoring method on low dose CT scans (7). This has resulted in some visual semi-quantitative score methods used in clinical practice, but there is no uniform method. Shemesh et al. (10) used Ordinal Scoring to predict the cardiovascular death in smokers and found a CAC score ≥4 was a significant predictor of cardiovascular death (HR 4.7, 95% CI: 3.3–6.8; *P* < 0001). Shao et al. (11) compared the Agatson scores to the non-gated chest CT for visible or no visible CAC and found similar predictive power for non-fatal MI and all-cause mortality. The non-gated chest CT can provide additional diagnostic information in addition to the lung parenchyma (12).

Kirsch et al. (6) showed that a Weston score > 7 is comparable to an Agatston score > 400 with sensitivity of 100%, and specificity of 98%. Bhatt et al. (13) indicated that the Weston score may have performed better than the Agatston score in predicting incident cardiovascular disease over 5 years of COPD patients, because the density of calcification is more weighted in the Agatston score than volume. However, coronary calcification volume is potentially a stronger predictor of cardiac disease (14). Hence the Weston score method may have some advantages from a technical standpoint. In our study, we found that the Weston CAC score was an independent predictor of major cardiovascular events and all-cause mortality. A CAC total score ≥5 was the optimal cutoff value for predicting MACEs. The results proved the predictive value of Weston CAC score for long-term prognosis. It may provide important coronary artery information for clinicians to intervene early and reduce long-term adverse cardiac events.

We found that the LAD artery was the most frequently diseased vessel in patients with asymptomatic CAD and subsequent MACEs, which correlated with the results of Matthew et al. (15). These findings suggest that clinicians should pay attention to the severity and specific locations of coronary calcification in addition to the traditional risk factors when managing patients beyond hospitalization (16, 17). We particularly want to point out that our data indicated significantly increased MACEs in patients with coronary artery calcium and diabetes, emphasizing the importance of primary prevention using statin drugs as strongly recommended by the current guidelines to start moderate-intensity statin therapy “in patients 40–75 years of age with diabetes mellitus and LDL-C ≥70 mg/dL (≥1.8 mmol/L)” (18). A positive coronary calcium scan results should provide clinicians an ideal window to implement guideline driven medical therapy and avoid missed opportunities treating this high-risk population.

It is worth to mention that in our study population, patients with zero calcium score had very low cardiac death rate during follow-up and only one died because of cardiac etiology. This corresponds to the MESA data showing that a coronary artery calcium score of zero resulted in the greatest downward shift in estimated CAD risk (19, 20). Clinicians can quickly discharge patients with zero calcium score and negative troponin results in their decision-making process for patients' safety while minimizing the unnecessary and costly test or procedures.

There have been numerous research articles published in the last decade about using CT CAC imaging for CAD risk stratification in diverse patient populations such as primary prevention, lung cancer screening, diabetic patients, patients with lung disease, and breast cancer. Our study took a different approach of comprehensive clinical data acquisition and long-term longitudinal follow up when analyzing the non-contrast chest CT results of emergency room or hospitalized patients for any reason scan. By using the semi-quantitative Weston method for coronary calcification, clinicians in the ED, hospital and outpatient clinics can quickly obtain highly relevant information to guide patient triage and management. With the wide utilization of non-contrast chest CT imaging, and very limited insurance coverage of dedicated CT CAC scoring, it is of important clinical benefits for radiologists to routinely report the CAC on the chest CT examination without additional testing and provide classification of the calcium burden as mild, moderate and severe degree as supported by the expert consensus statement from the Society of Cardiovascular Computed Tomography in 2017 and 2018 (21, 22). CAC scores without CT coronary angiography maybe very useful as a risk mark in patients with chest pain or other cardiac symptoms in the emergency room. But its role in evaluating patients with non-ST elevation acute coronary syndrome is limited since these patients usually require coronary intervention, and may have soft plaques without much or any coronary artery calcification. Puchner and colleagues reported that total CAC burden was associated with ACS but segmental CAC was not associated with culprit lesions (23). The main limitations of our study are: while our case mix represented most patients, who presented for unenhanced non-gated chest CT in the emergency room, hospital, or outpatient with subsequent hospitalization due to any possible cardiopulmonary etiologies; it was a single center retrospective cohort; and all our patients were referred by treating physicians for suspected cardiopulmonary diseases. Weston method of CAC scoring was successful in 89.6% patients screened due to technical difficulties such as motion artifact. Our sample size is also relatively small compared to many multicenter studies or national and international registries. We want to remind the readers to avoid the generalizability of interpreting our results. Future research should focus on predictive value for a larger patient population to maximize the clinical utility of chest CT evaluation.

## Conclusions

The Weston method for assessing coronary artery calcification can be easily performed after non-gated chest CT studies. Our long-term follow up showed relevant clinical data integration for the identification of silent CAD, which may help in possibly early initiation of therapies for primary prevention of cardiac events, especially in patients with LAD coronary artery calcification and total coronary artery calcification score ≥5. Our data may also have clinical implications for appropriate patient follow up utilizing available CAC data after hospital discharge.

## Data Availability Statement

The raw data supporting the conclusions of this article will be made available by the authors and governed by the HIPAA rules.

## Ethics Statement

The study was approved by the institutional review board of Stamford Hospital (Quorum Review Institutional Review Board QR# 32130) as a retrospective chart review with HIPAA and informed consent waiver as part of the approval.

## Author Contributions

LC and DH designed the study and drafted the manuscript. AV, JR, PD, and JB collected the data. MB, ME, MG, SR, and AF were involved in data and manuscript review, follow-up, and verification. LC and BS analyzed the data. DH approved the final version of the manuscript. All authors contributed to manuscript revision, read, and approved the submitted version.

## Conflict of Interest

The authors declare that the research was conducted in the absence of any commercial or financial relationships that could be construed as a potential conflict of interest.

## Appendix

**Table A1:** CAC scores in patients with major adverse cardiovascular events.

	**Cause**	**Total CAC score**	**LAD score**	**LM score**	**LCX score**	**RCA score**
1	Non-fatal MI	12	3	3	3	3
2	Non-fatal MI	12	3	3	3	3
3	Non-fatal MI	4	3	0	1	0
4	Non-fatal MI	12	3	3	3	3
5	Non-fatal MI	9	3	3	0	3
6	Non-fatal MI	11	3	2	3	3
7	Non-fatal stroke	1	1	0	0	0
8	Non-fatal stroke	0	0	0	0	0
9	Myocardial revascularization	5	3	0	0	2
10	Myocardial revascularization	9	3	1	2	3
11	Cardiac death	8	3	1	2	2
12	Cardiac death	6	3	0	0	3
13	Cardiac death	11	3	2	3	3
14	Cardiac death	10	3	2	3	2
15	Cardiac death	11	3	3	3	2
16	Cardiac death	12	3	3	3	3
17	Cardiac death	6	3	0	0	3
18	Cardiac death	9	3	0	3	3
19	Cardiac death	10	3	3	3	1
20	Cardiac death	10	3	2	2	3
21	Cardiac death	12	3	3	3	3
22	Cardiac death	12	3	3	3	3
23	Cardiac death	12	3	3	3	3
24	Cardiac death	3	3	0	0	0
25	Cardiac death	11	3	3	3	2

*LM, Left main trunk; LAD, Left anterior descending artery; LCX, Left circumflex artery; RCA, Right coronary artery*.
